# Training healthcare professionals to be ready for practice in an era of social distancing: a realist evaluation

**DOI:** 10.1007/s10459-023-10297-w

**Published:** 2023-12-08

**Authors:** Janet Lefroy, Jessica Bialan, Alice Moult, Fiona Hay, Claire Stapleton, Jessica Thompson, Kate Diggory, Nageen Mustafa, Julia Farrington, Sarah A. Aynsley, Simon Jacklin, Adam Winterton, Natalie Cope

**Affiliations:** 1 Clinical Education Centre RSUH, Keele University School of Medicine, Newcastle-under-Lyme, ST4 6QG UK; 2 Impact Accelerator Unit, Keele University, Newcastle-Under-Lyme, ST5 5BG UK; 3 School of Allied Health Professions, Keele University, Newcastle-Under-Lyme, UK; 4 Keele University School of Pharmacy and Bioengineering, Newcastle-Under-Lyme, UK; 5 Keele University School of Nursing and Midwifery, Newcastle-Under-Lyme, UK

**Keywords:** COVID-19 pandemic, Undergraduate healthcare students, Clinical preparedness, Placements, Clinical skills

## Abstract

**Background:**

Programme changes due to the COVID-19 pandemic have impacted variably on preparation for practice of healthcare professional students. Explanations for such variability need exploration. The aim of our study was to understand what clinical learning, whilst under socially distanced restrictions, worked and why (or why not).

**Methods:**

We conducted a realist evaluation of the undergraduate healthcare programmes at one UK university in 2020–21. Initial programme theories to be tested in this study were derived from discussions with programme leads about the changes they implemented due to the pandemic. Study participants were students and teaching faculty. Online interview transcripts were coded, identifying why interventions had worked or not. This resulted in a set of ‘context-mechanism-outcome’ (CMO) statements about each intervention. The initial programme theories were refined as a result.

**Results and discussion:**

29 students and 22 faculty members participated. 18 CMO configurations were identified relating to clinical skills learning and 25 relating to clinical placements. Clinical skills learning was successful whether in person, remote or hybrid if it followed the steps of: demonstration—explanation—mental rehearsal—attempt with feedback. Where it didn’t work there was usually a lack of observation and corrective feedback. Placements were generally highly valued despite some deficiencies in student experience. Being useful on placements was felt to be good preparation for practice. If student numbers are to expand, findings about what works in distance learning of clinical skills and the value of various modes of induction to clinical workplace activity may also be relevant post-pandemic.

**Supplementary Information:**

The online version contains supplementary material available at 10.1007/s10459-023-10297-w.

## Background

Healthcare education has been impacted by the COVID-19 pandemic and its resulting social distancing requirements. With increasing hospital admissions, high death rates and critically stretched healthcare resources, governments around the world enforced social distancing and self-isolation to reduce viral spread. While this had implications across many sectors, education both within schools and higher education was greatly affected in the UK and globally, not least for the training of healthcare professional students.

There were adaptations to healthcare education made as a result. Different innovations or approaches have been taken with variable outcomes. When UK higher education programmes (including healthcare) sent students home for online delivery of learning in March 2020, some final year healthcare students were offered the opportunity to move in the opposite direction to support the workforce by taking up intermediary clinical roles with new supplementary roles created for more junior students. Many did, if they were not clinically vulnerable or providing home schooling and care for family, including 28,108 student nurses and student midwives and 4662 interim foundation doctors (Health Education England, 2020a, 2020b). As the pandemic continued longer than anticipated, some senior healthcare students were allowed to recommence placement while junior years remained online. This move was in keeping with guidance from governing councils who emphasised the need to restart clinical education with particular emphasis on ensuring preparedness of final year students while first- and second-year students were to continue studying online (Medical Schools Council, 2020; Medical Schools Council & General Medical Council, 2021). There was disparity between the healthcare disciplines in the extent to which they continued placements and offered deployment or moved students out of the clinical workplace and into alternative learning activities. Nursing students have seen the least disruption with one cross-sectional study reporting over half of respondents continuing with no changes and only 36% having to relocate to alternative placements (Ulenaers et al., 2021). While medical and nursing governing bodies encouraged student deployment, a joint report from Health Education England and the Royal Pharmaceutical Society discouraged this in favour of students focusing on their studies (Health Education England, 2020a, 2020b).

With clinical placements curtailed, innovations to supplement clinical learning at a distance evolved. Of these, simulation—already a well-received and popular tool across the healthcare sciences—was expanded by some programmes to give students clinical exposure without patient interaction (Almohammed et al., 2021; Jiménez-Rodríguez et al., 2020; Kasai et al., 2021). New technologies were trialled for clinical learning, for example, telehealth student-patient consultations and the use of a modified reality (MR) lens which allowed medical students to remotely participate in ward rounds (Bala et al., 2021).

We reviewed previous studies which indicate that the programme changes due to the pandemic have impacted variably on preparation for practice of healthcare students. Articles published between March 2020 and May 2021 indicate that students missed a significant portion of their clinical training but expressed conflicting opinions regarding their level of preparedness.

Evidence of negative impact included feeling less prepared to start work as a doctor (Choi et al., 2020), dissatisfaction with the inflexibility in requirements for competency sign-offs (Ulenaers et al., 2021) and concern that in certain specialties there were few opportunities for practicing skills. This gap in clinical skills represents a potential challenge to preparedness or implications for future placements if remediation is required. Some students also felt disadvantaged by the move to online learning (Chesterton et al., 2022).

Contradictory evidence showing positive impacts has also emerged, with some students feeling that their training during the COVID-19 pandemic was adequate (Brown et al., 2021). Telehealth innovations were appreciated in the circumstances as a (limited) opportunity to work with patients and healthcare professionals (Bautista et al., 2020; Kopp et al., 2021; Yang et al., 2020). Although deployment of nursing and medical students to support the workforce was challenging for some, especially initially (Goni-Fuste et al., 2021; Griffin & Riley, 2022), in other studies volunteering within the workforce was highly beneficial for aspects of clinical preparedness, benefitted multidisciplinary relations and promoted patient centred behaviour (Ali et al., 2021; Byrnes et al., 2020; Chawłowska et al., 2021; Choi et al., 2020; Coster et al., 2022; Kuliukas et al., 2021; Nolan & Owen, 2021; Siqueira et al., 2022; Tempski et al., 2021; Ulenaers et al., 2021). Nursing students valued the opportunity to decide for themselves whether they would like to continue placement, defer or be deployed (Ulenaers et al., 2021). Radiography students were offered the opportunity to take up either early registration or a paid assistantship prior to registration; those who took part in both expressed a number of benefits to clinical preparedness including teamworking skills, resilience and additional experience under supervision (Cushen-Brewster et al., 2021).

What is missing in the research into the impact of training during the COVID-19 pandemic on healthcare students is explanations of why some are confident, satisfied and thriving while others are not. The disparate effects of COVID-19 on clinically vulnerable or mature students needs exploring. We found few original research articles reviewing the universal effects of COVID-19 across students of multiple healthcare professions and few studies of the impact of COVID-19 on clinical preparedness and education of pharmacy, physiotherapy and radiography students.

It is important that we understand how effective the learning strategies employed during the COVID-19 pandemic have been for three reasons. Firstly, healthcare graduates who trained in 2020–2022 may need educational gaps filling. Secondly, we should be better prepared for another pandemic. Thirdly and possibly most importantly, innovations such as hybrid learning of skills and virtual placements are being continued after the pandemic to aid inclusivity through ease of modes of access and to train increasing numbers of healthcare professional students. Through understanding what strategies work for who, and why, it may be possible to train successfully in a more flexible working environment. The aim of our study was therefore to understand how we can best train healthcare professionals to be clinically ready in an era of social distancing. Specifically, we aimed to understand what teaching and learning of clinical skills and activities, whilst under socially distanced restrictions, worked for students and faculty and why (or why not).

## Methodology and methods

To explore why socially distanced healthcare training works well for some students and not so well for others, a realist approach to the research question was chosen. The justification for this is that realist evaluation investigates how a complex human system works and what aspects of the context impact the outcome positively or negatively. When evaluating healthcare training in a pandemic, context needs to be considered in just such a specific way because the likelihood is that a similar educational intervention might have a different outcome when conditions are different. In realist evaluation an iterative approach is used to test, develop and refine initial theories about how the programme works (Pawson & Tilley, 1997; Wong et al., 2016). Realism acknowledges that aspects of a context shape the way individuals respond to an intervention. The intervention will therefore produce various outcomes when implemented in different contexts through a fairly predictable set of human reactions (mechanisms). Realist evaluation asks ‘what works for whom, under what circumstances, and how’ and is often expressed in the formula C + M = O, where C = context, M = mechanism and O = outcome. Contexts are defined as the conditions in which an intervention operates, predominantly socio-cultural but not exclusively. Mechanisms are the reasons for actions that people take in response to the intervention. For example, a student whose parent has recently died of COVID-19 (C) may find seeing the patients (C) on an ICU placement (intervention) triggers painful memories (M) with a negative impact on wellbeing and their ability to learn (O).

The study was conducted in the health faculty at Keele University, UK which is a rural campus university 3 miles outside the city of Stoke on Trent and the busy urban University Hospital of the North Midlands where most of the healthcare student placements are situated. The initial programme theories to be tested in this study were derived by the core research team meeting with the leads of each undergraduate pre-registration healthcare professional programme at Keele University (nursing and midwifery, medicine, pharmacy, physiotherapy and radiography). Before meeting we asked them to consider the question ‘How is our curriculum going to get our graduates clinically ready in the socially-distanced learning environment?’. We then discussed with them the strategies (interventions) they were adopting and how (mechanisms) these strategies were intended to produce the required graduate readiness (outcomes).

The research team also drew on the governing body directives for medicine (General Medical Council, 2018), pharmacists (General Pharmaceutical Council, 2011; Future Pharmacists Standards for the Initial Education and Training of Pharmacists, 2011), physiotherapists (HCPC, 2013a), radiographers (HCPC, 2013b) and nursing and midwifery (Future Nurse: Standards of Proficiency for Registered Nurses, 2018; NMC, 2019) along with the Emergency Standards for Nursing and Midwifery Education (NMC, 2020) introduced in March 2020, some phased out Sept 2020, some reintroduced Jan 2021 and finally withdrawn Sept 2021. The initial programme theories are described before the results section. Initial programme theories about online group and self-directed learning were also developed and tested with participants and are the subject of a companion study.

### Data collection

The study data was interview transcripts containing explanations from individuals of what worked and why in their experience of their undergraduate healthcare training programme since the onset of the pandemic. Participants were students and teaching faculty from each of the schools in the Faculty of Health at Keele University who were purposefully sampled to be interviewed. Faculty were identified by their head of programme and were invited by personal email. Clinical student year groups were contacted via email lists for each programme and respondents were accepted up to a limit proportionate to the size of their cohort.

Researchers were trained in realist interviewing and coding in June 2020. Interviews were conducted via Microsoft Teams between November 2020 and October 2021 by researchers who were from a different programme from the participants they interviewed. Interviews lasted approximately 40 min and were audio recorded. A topic guide was used to guide the interview (see Online Appendix 1), informed by the initial programme theory. Audio recordings were transcribed verbatim using an approved transcription service. All identifying data was removed.

This study was approved by the University ethics panel on 22.6.20 ref. KR-200021.

### Analysis

Two researchers (the interviewer and a researcher from the participant’s own school and therefore able to understand programme-specific contextual comments) independently coded each transcript using realist coding in which the coder is looking for ‘context-mechanism-outcome configurations’ (CMOcs) relating to the interventions in the programme (Pawson & Tilley, 1997). Text was coded in the transcript word documents using comment boxes in the margin of highlighted chunks of text for initial CMO annotation, the two coders each working on a clean copy of the text. This resulted in a set of CMOc statements for each intervention. These statements with supporting quotes were exported into an Excel spreadsheet for further analysis. Each statement was colour-coded according to whether the intervention was working or not. Where an intervention was described as working for some individuals in some contexts but not in others, it was coded with both colours.

The final phase of the analysis consisted in determining which CMOc(s) offer the most robust and plausible explanation of the observed pattern of outcomes for each intervention. CMOcs were grouped by JL into the initial programme theory they were being used to test, and sub-divided into similar topics, generally by the intervention being referred to. The initial CMOcs from individual scripts were consolidated by JL and NC by discussion into overarching CMOcs containing what worked or didn’t in various contexts. Two researcher meetings discussed and refined these CMOcs. For each of the final CMOcs, one or two illustrative quotes across the healthcare programmes were selected by JL and NC. These overarching CMOc statements refined the initial programme theories.

### Initial Programme Theories developed with the heads of programmes (see also schematic representation Fig. 1)

**Fig. 1:**
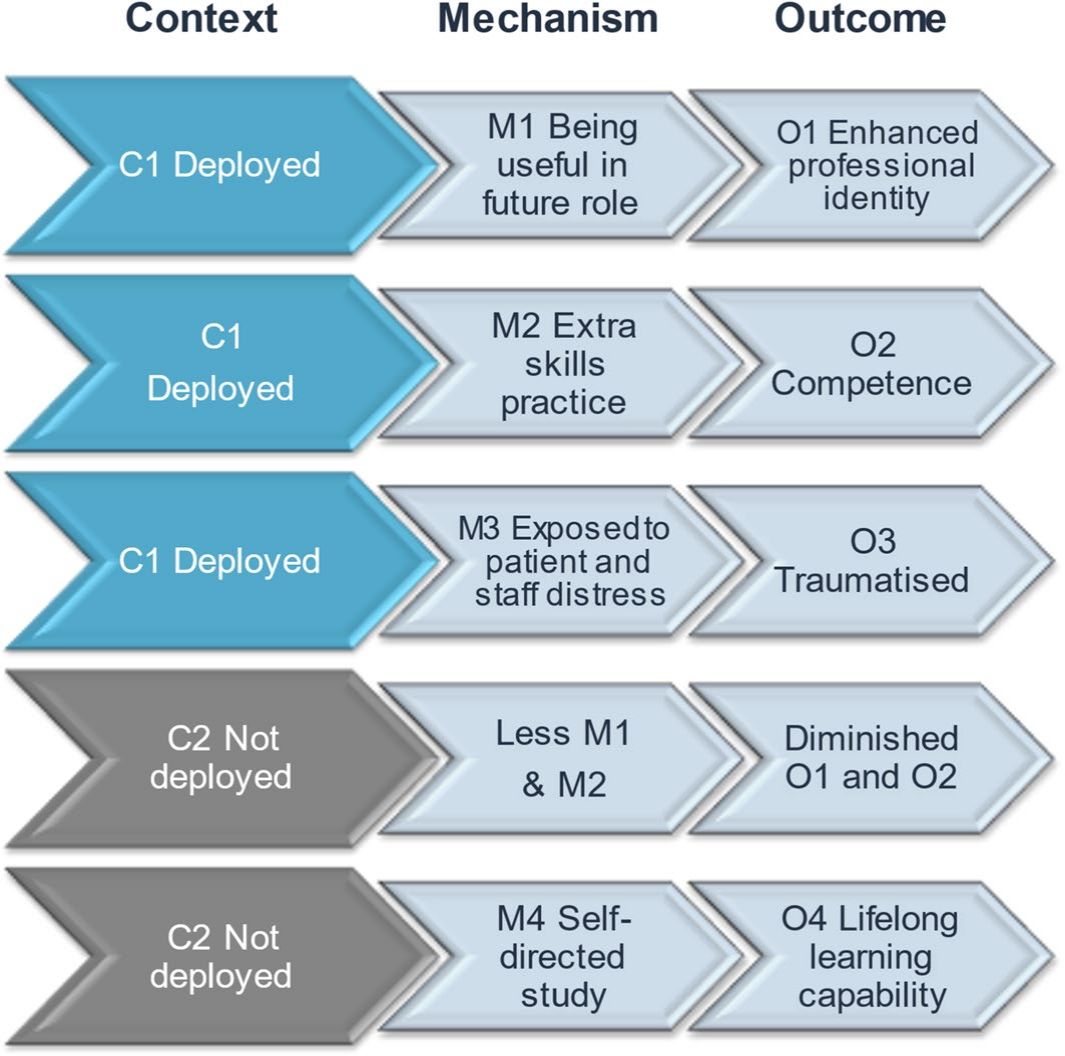
Schematic of the Initial Programme Theories 3 and 4 relating to NHS deployment/volunteering by students—displayed as Context-Mechanism-Outcome (CMO) configurations

Each programme aimed to train and graduate fully competent healthcare professionals through classes, self-directed learning, simulation, workplace learning and reflection following educational theories as they did pre-pandemic (Taylor & Hamdy, 2013) although in different proportions and with modifications.

In the **macro-context** of.

- Fear of COVID-19, social distancing rules, PPE in clinical practice and periods of self-isolation
- Regulatory Body requirements altering in some courses (reduced requirement for total hours to qualify for nursing and physiotherapy, but no reduced requirement for practical procedure sign-offs)

The modified undergraduate health professional programme activities **(C)** will work differently **(O)** for *various groups of students* **(C)** by the following learning processes or influences on student thinking and actions **(M):**

1. Clinical skills learning
  Increased clinical skills learning and practicing by simulation, remote where possible and in-person when necessary (C), was predicted to mitigate for some of the lost clinical placement practice and would also reduce COVID-19 transmission to and from the clinical workplace. The traditional clinical skills learning mechanisms of observation, repeated deliberate practice and feedback (M) (Ericsson, 2004; Giacomino et al., 2020) were expected to produce clinical competence (O) and self-efficacy (O).
  Social distancing in clinical skills classes (C) meant smaller groups(C) which would offer more opportunity for tutor observation and feedback (M), fixed student groups(C) which could enhance peer learning (M), but briefer classes(C) which might restrict deliberate practice (M), wearing PPE(C) with no refreshment breaks(C), which were expected to disrupt learning by discomfort (M). The outcomes were hoped to be equivalent to previous learning on balance but this was uncertain.
2. Clinical placements
  Students were sent home from March to July 2020 (C). On their return, student numbers on each placement were reduced for social distancing (C). Programme leads theorized that when students were present, due to their small numbers they might get to do more (M), act more as team members (M) and take more responsibility (M), resulting in enhanced development of professional identity (O) and self-efficacy for clinical tasks (O).
    It was predicted that senior students (C) had priority and would therefore receive these benefits, conversely junior students (C) had reduced placement exposure and would miss these benefits (O).
    Student risk assessment by Occupational Health scored them low-medium-high risk for severe complications should they catch COVID-19. Medium and high risk students were excluded from some or all clinical placements (C) so would also lose these learning opportunities (O).
  There was some remedial placement provision (C) but students who missed placement experience might have gaps in their training (O).
  Reduced socializing in the workplace (C) was expected to affect all students but especially those just starting clinical learning, as there would be less than usual learning by the informal role modelling of near peers and senior colleagues(M). The outcomes predicted were delayed or distorted development of professional identity (O).
  New ways of communicating remotely with patients and family members were to be promoted in the curriculum (C) and practiced by students on placements supported by tutors (M) to provide equivalent and currently-appropriate consultation skills training (O).
  3. Deployment/Volunteering
    Health Education England asked schools to encourage emergency student contracts and NHS volunteering (these were entirely voluntary, not for all students and not counted as placements). Where students were deployed or employed as healthcare workers (C) this was expected to substantially mitigate the above impacts of reduction in placement exposure (M) and to enhance professional identity and team-awareness (O) COVID-19 awareness (O) and clinical skills (O). The students who were not able to be deployed/employed because they were clinically vulnerable (C) or had childcare responsibilities (C) or were junior (C) were expected to be most severely affected by being de-skilled (M) and suffer low self-efficacy (O) but might mitigate this by doing more self-directed learning (O).
    4. Exposure at work and in the family to people very ill and dying with COVID-19 (C) was expected to be traumatizing (M) for some students with effects on mental health and resilience (O).

An illustrative schematic of how Initial Programme Theories can be represented visually showing the relationship of context to outcome via expected mechanisms is offered in Fig. 1.

The scope of the evaluation was to test these theories in the experience of participants. Interviewers were familiarized with the expected learning mechanisms and outcomes for the programme about which they were interviewing and could explore in depth whether the theory was borne out in practice. In analysis, the evaluation framework was based on the initial programme theories.

## Results

We recruited 29 students and 22 faculty members for interview to help us to test the initial programme theories (Table 1).

**Table 1:** Study participants by status (student or faculty) and by programme of study

Programme	Students	Faculty	Annual intake of students
Adult Nursing	5	2	130
Children’s Nursing	1	1	24
Mental Health Nursing	2	2	40
Learning Disability Nursing	2	1	14
Midwifery	1	2	33
Physiotherapy	4	3	105
Radiography	2	2	30
Pharmacy	5	5	140
Medicine	7	4	175
Total	29	22	691

The CMOcs extracted from our data which are relevant to readiness for clinical practice are presented below and in online Appendix 2 where they are illustrated by one or more quotes which are drawn from across the healthcare programmes. For ease of understanding causation in each CMOc statement, the intervention is identified in the statement, and the causative elements in both the intervention and the wider pandemic context are denoted by (C) to make clear what is causing the outcome(s) (De Weger et al., 2020). Mechanism is denoted by (M) and outcome by (O). Participant ID after each quote indicates the participant’s profession, whether they are faculty or student with year of study eg NA23 is a student of adult nursing in their second year and is the third to be recruited.

### Findings about learning clinical skills

All participants gave explanations of how aspects of clinical skills learning worked (or didn’t work) for them and student colleagues, condensed into 18 CMOcs which can be viewed in full in Online Appendix 2. We have selected the following key CMOcs for discussion:

#### How learning skills theory and watching demonstrations online, then practice in person worked: (CMOc 1.1)

In the macro-context of COVID-19 restrictions requiring reduced in-person activity (and for pharmacy students no placements) the intervention was to provide pre-learning videos and audio recordings for basic skills learning then (shorter than previously) classroom simulated clinical practice and/or simulated practice at home.

This worked for some skills C: theory-heavy, less complex tasks

M: Learning by observation, retention and retrieval

O: Students came better prepared to skills classes than pre-pandemic

But for other skills C: more technical, less theory

M: the mechanism of retention didn't operate

and if C: theory was learned a long time before the skill practice

M: the mechanism of retrieval didn't operate

C: for students who had no possibility to practice at home

also C: for students not getting practice with patients with signs

M: (missing in this context) practice - learning by doing

O: Skills not retained if not consolidated by repetitive practice

#### How skills learning moving from classroom to placement worked (CMOc 1.2)

Nursing students continued to spend a lot of time on placements but due to COVID-19 had to reduce in-person classes so some skills were allocated to be learned online / by written theory rather than in skills classes (Intervention) then straight to clinical placement for practice.

If: C: the placement provided practice and feedback.
  M: learning by practicing the technical and communication aspects of the skills
  M: the personal reinforcement of feedback
  O: skills were acquired safely (the intervention was successful)
BUT if: C: Placements didn't support first attempts.
  M: (Missing in this context) safe practice
  O: students feeling under-prepared so were anxious when performing skills on placements and may not have done them correctly

#### How skills classes in person worked when socially distanced with no touch rules for demonstration of skills (CMOc 1.3)

For physiotherapy and pharmacy students social distancing rules meant that staff were unable to demonstrate on students. Instead, students were permitted one partner to practice with.

C: Staff unable to demonstrate on students

C: students had to observe from a distance

M: (Missing in this context) effective demonstration

M: (Missing in this context) hands-on correction

M: verbal feedback instead

O: Ineffective learning experience—less feedback obtained, less confident in practical skills.

#### How skills practice at home worked (CMOc 1.4.3)

This worked if students lived with other students on the same course and had already learned the skills:

C: living with other students (or not)

M: repetitive clinical practice and feedback (or not)

O: Hone technique and confidence about competence and getting through assessments (or not)

C: if the students had not already had (classroom) corrective feedback on a first attempt

M: (missing in this context) corrective feedback

O: Therefore didn't work for a new skill.

#### How consultation skills classes worked online (CMOcs 1.6.1 and 1.6.2)

For medical and pharmacy students, online consultations and handovers worked well. The observing tutor and class could become ‘invisible’ by switching their cameras off which enabled the interacting students to imagine the interaction was one-to-one. This was less daunting for students with lower confidence levels.

C: observers instructed to have cameras off during roleplay

C: students with different levels of confidence

M: able to do effective simulated handovers, able to imagine a patient consultation is one-to-one

O: Worked well including for the less confident. Preparedness to do handovers, to consult with patients

Online didn’t however feel quite authentic when simulating a sensitive in-person interaction remotely in order to reduce COVID-19 risk for Simulated Patients:

C: discussing sensitive topics (eg resuscitation decisions) via video link

M: Simulated Practice – first time

O: really useful to have the first experience, (feeling prepared for the real thing)

O: BUT Online didn’t feel quite authentic

#### How high-fidelity (authentic) simulation worked (CMOcs 1.7.1. to 1.7.3)

Various high-fidelity simulations already being used for in-person simulations in radiography (a simulated Xray facility) and medicine (in-person SIM man, simulated on-call) were augmented by virtual simulations in pharmacy (a virtual patient and simulated pharmacy), medicine (Oxford Medical Simulator) and radiography (a virtual control panel with shared controls via the online platform). These simulations worked for junior (radiography) students who were more prepared when starting placements than students who learned theory only online. They also worked to make senior students better prepared to take responsibility by simulated practice in their ‘zone of proximal development’ (Vygotsky, 1986).

Intervention: Learning clinical skills in simulation in person

C: High fidelity sim Xray facility, SIM man, Simulated on-call

M: Authentic experience and practice

M: learning from feedback

M: learning decision-making

O: Juniors are more prepared for placements. Seniors are more prepared to take responsibility

Virtual simulation for medical and pharmacy students is less authentic than in-person simulation as it works by clicking buttons rather than speaking and laying hands on the patient, choosing from set questions rather than asking the questions you want to. Despite this, it was appreciated by pharmacy students (who were getting no other patient contact). It was deemed less educational than in-person SIM for medics but good to reinforce learning.

Intervention: virtual reality simulators

C: Pharmacy students getting no other patient contact

C: Medical students getting in person SIM and real patient contact

M: not wholly authentic practice

M: corrective feedback

O: Appreciated by pharmacy students. Less educational than in person SIM for medical students but good to reinforce learning

C: for radiography students staff can share screen online / give others control of their screen

M: setting up an Xray is same as authentic practice

O: works well for radiographers.

Simulation was noted not to authentically match the workplace noise, smells and pressures.

C: Simulated workplace is quiet.

C: Actual workplace distractions such as noise and pressures

M: shock when exposed to reality

O: Junior students may be partly prepared for placements by simulation but not completely. Pharmacy and physiotherapy students who missed placements will need more support in their first jobs to find their feet

### Findings about clinical placements

The programme interventions to provide safe clinical experience for healthcare students were multiple and altered with time, responding to the changing risk levels and to the way the local healthcare trusts were working. The data contained 189 explanations from participants about what aspects of in-person and virtual clinical placements they felt had worked or not to prepare them or their student colleagues to be ready for their future clinical work and why. The commonly-occurring (robust) and plausible explanations are presented in Appendix 2 in twenty five CMOcs 2.1.1 to 2.6.4. The key CMOcs we wish to highlight for discussion are:

#### Placements were still expected to provide the required clinical experience despite COVID-19. How this worked: (CMOcs 2.2.1 to 2.2.3)

The patient mix, clinical activity and restriction of student access altered on some placements more than others.

C: Where placements were maintained (not all were comparable)

M: provides a variety of opportunities for skill practice

O: clinically prepared.

In the context of unchanged assessed intended learning outcomes and regulatory body requirements, the pressure of getting 29 nursing proficiencies signed off was a concern to mental health and learning disability nurses whose placements were not providing these opportunities. For schools not permitted to send students on placements (pharmacy and physiotherapy) the extent of missing clinical experience and assessment opportunities and lack of confidence in using their knowledge was considerable:

C: COVID-19 disruption of placement allocations causing rapid change and lack of predictability

C: Regulatory body requirements unchanged

C: Some courses not permitted to send students on placements (pharmacy and physiotherapy)

M: (reduced in this context) clinical experience and assessment opportunities

O: lack of confidence in using their knowledge

O: some will require remediation (possibly some after qualification)

#### Students were made useful in the workforce (when on placement and also by encouraging deployment and volunteering). How this worked: (CMOcs 2.3.1 to 2.3.4)

C: the duties expected of the graduate (unchanged in the pandemic) and the need for graduates to be work-ready in the pandemic

M: doing the tasks of the healthcare professional (rehearsal)

M: becoming part of the team

M: theory put into practice, tailored to the patient

M: constructive feedback from senior (checking and rectifying)

O: Consolidated knowledge (and reasoning) and decision-making

The outcome for deployed students was to become more confident and skilled when they joined the workforce after graduation, especially if they had been deployed in the location where they were about to have their first job. Those students who did other healthcare jobs paid or as volunteers also became confident and generally satisfied training requirements although there was some competition between their educational needs and the workforce gaps they were filling.

C: Those students who did healthcare jobs

M: gain exposure

M: are given and take responsibility as healthcare team members

O: they feel confident

O: and earn money

O: and satisfy training requirements

O: and support the health service

C: Need for students to help during the pandemic

C: the need to still gain proficiencies to qualify

M: Clinical skills learning (competing with) M: Responsibility—helping in the pandemic.

O: less easy to gain all required proficiencies.

Those who didn't (vulnerable, having caring responsibilities, lacking opportunity) were expected to lack confidence.

C: Those students who couldn't be deployed or volunteer

M: (lacking) clinical experience

O: they will feel daunted in their first jobs.

#### How placement induction worked for junior students: (CMOcs 2.4.1 and 2.4.2)

New and detailed inductions of students familiarizing them with the COVID-19 clinical environment generally worked well to prepare them for what would be different on placements.

C: the clinical workplace is unfamiliar to new students especially since social distancing and PPE introduced

C: COVID-19 risk – some anxious students

M: familiarisation discussion/reading

M: role modelling

O: students better prepared for placements

O: motivation for workplace learning.

However, for physiotherapy students who had no prior clinical exposure, learning about clinical theory didn’t make sense:

Intervention: Theory of clinical practice taught in Y1 and applied in placements Y2

C: For students who have no prior clinical exposure

M: 'clicks' (making sense of theoretical learning) when they meet patients face to face

O: learning now has meaning

Their tutors commented that despite this, no more students than normal failed early years assessments.

#### How virtual placements worked: (CMOcs 2.5.1 and 2.5.2)

Pharmacy, physiotherapy and radiography students (and to a small extent medical students) had substitute virtual (group discussion with clinicians) placements to provide simulated clinical decision-making practice and exposure to clinical scenarios. They were felt to have some value but were only partial preparation.

C: COVID-19 risk and remote students

Intervention: Virtual placement

M: Simulated clinical decision-making practice

M: Exposure to clinical scenarios

M: (lacking in this context) hands-on nature of real practice

O: Junior students are better prepared for placement practice of those topics which have been covered

O: Only partial preparation. Senior students are lacking hands-on skills - pharmacy, radiography and physiotherapy graduates will have gaps needing support. International students likely to have missed more in person placements

Also students who were struggling were thought to be going undetected on virtual placements.

C: Students are not working closely with clinical supervisors in clinical practice

M: Tutor misses noticing how students work in a clinical setting

O: Students who are struggling go undetected and don't get help.

## Discussion

Our participant healthcare undergraduates and their faculty have provided rich explanations of what has worked and not worked in their training during the COVID-19 pandemic to make them ready for practice. We have used these explanations to confirm and develop our initial programme theories about how the adjustments to clinical skills learning and placement activities were expected to work. These prior theories were largely supported. Clinical preparedness in a pandemic is however a complex and still-developing area of educational theory, for which we are not claiming to have produced a complete set of final programme theories. Rather, our study’s main findings develop several areas of theory.

**Fig. 2:**
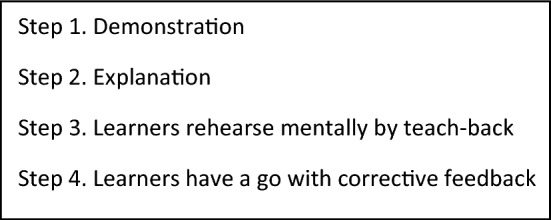
Payton’s four step process of learning a skill

Firstly, we were able to confirm that learning basic technical skills in a socially distanced way worked just as it does in person when it followed the four-step process of learning a skill by watching a demonstration; getting an explanation of how the skill is performed correctly and why; explaining this back step by step while the teacher or another learner follows their instructions; and finally performing the skill themselves and getting feedback (Giacomino et al., 2020) (see Fig. 2). From faculty and student explanations of what worked and didn’t work in the many adjustments to socially-distanced skills teaching, a theory of how this worked best was developed.

The demonstration could be by (high enough quality) video unless it was for skills involving manual handling prevalent in physiotherapy when students benefit from acting as a model to feel the patient’s experience of being handled. The step 2 explanation could also be delivered remotely, and the benefit of step 1 and 2 being remote was that this enabled learner pacing of delivery and re-running. What didn’t work was too long a gap between step 1 and 2 and step 4. Also if step 4 comprised a single simulated attempt only, or if step 4 was directly on a real patient this made the learner feel anxious and unprepared. The main explanation for this was the omission of corrective feedback on first attempts. Student participants reported discovering that they were performing skills incorrectly (with the potential for future dangerous practice) or lacked confidence in their performance.

Peer physical examination is held to have many educational benefits, including learning normal anatomy, understanding the patient’s experience, improving confidence and getting corrective feedback (Hattingh & Labuschagne, 2019). In the context of the COVID-19 pandemic, this was a successful way of learning systems examinations for medical students but was again inadequate for physiotherapy students. This was because when tutors were not permitted to touch students due to social distancing, student ‘bubbles’ were poor substitutes for tutor demonstration and could be disrupted by frequent 2-week self-isolation spells as alternative pairings were not permitted. The demonstration and corrective feedback steps of the learning process were impaired.

Deliberate practice is known to be necessary for the acquisition of expert performance (Ericsson, 2004). What our findings highlight is the importance of close observation and corrective feedback at the initial stages of deliberate practice. Simulated clinical scenarios worked well for this when authentic enough as a representation of the clinical workplace as found in previous studies (Almohammed et al., 2021; Jiménez-Rodríguez et al., 2020; Kasai et al., 2021) but were unable to replicate everything that students felt they needed in order to be clinically prepared, so placement practice was also necessary. Paper/online cases with discussion didn’t work so effectively for developing clinical expertise because they didn’t involve performing the skills.

The key social theories of workplace learning such as situated cognition in communities of practice (Lave & Wenger, 1991; Wenger, 2000), and needing support for learning in the zone of proximal development (Vygotsky, 1986) were seen to still apply even when social distancing and fear of contagion were distorting the shape of the workplace community. Social interaction, observation and modelling of desired behaviours, a supportive learning environment and feedback were all important learning mechanisms. Students who felt in the way, or unsupported by clinical supervisors reported outcomes of low confidence and gaps in learning. The findings from participants’ experiences of clinical placements during COVID-19 may be relevant to ensuring preparedness in the context of any future similar pandemic. There are some explanations of why interventions worked or didn’t work which can be transferred to future healthcare professional training.

Another aspect of learning in a community of practice is legitimate peripheral participation (Lave & Wenger, 1991). Volunteering and helping during the pandemic was expected in initial programme theories to enhance professional identity and this was found to be the case. Participants explained how being useful on placements was good preparation for practice as being given responsibility triggered a transition in their thinking to a more professional and team-oriented approach. The same mechanisms are likely to have operated in other studies of pandemic volunteering and deployment (Ali et al., 2021; Chawłowska et al., 2021; Coster et al., 2022). Getting the job done was a challenge which caused them to draw on their knowledge and use decision-making processes which they could see they require after qualification. If there was checking and rectifying feedback from supervisors this felt like the best preparation possible and created a high level of confidence.

Participant explanations from junior students about the value of various modes of induction to clinical workplace activity may also be relevant post-pandemic. The clinical environment is quite alien but does not wait for the student to catch up and does not permit much asking of questions, so equipping students with some knowledge and basic skills enabled them to make better use of their first placements. As student numbers increase, placements in clinical workplaces will be fewer per student and are being supplemented by virtual placements. These can be oriented towards preparation for clinical placements, and the first clinical placement should be early to help the learning to ‘click’ and have meaning. The challenge to faculty in attempting to increase simulated practice to replace placements should be noted. Because of the constraints on space and clinical tutor numbers, the initial programme theory that increased clinical skills learning and practicing by simulation would mitigate for some of the lost clinical placement practice was not borne out in reality. Some programmes managed to maintain the same level of simulated practice as previously while others delivered less than usual due to social distancing rules and pandemic conditions. Going forward the context will be different but increasing simulation will need resourcing.

**Fig. 3:**
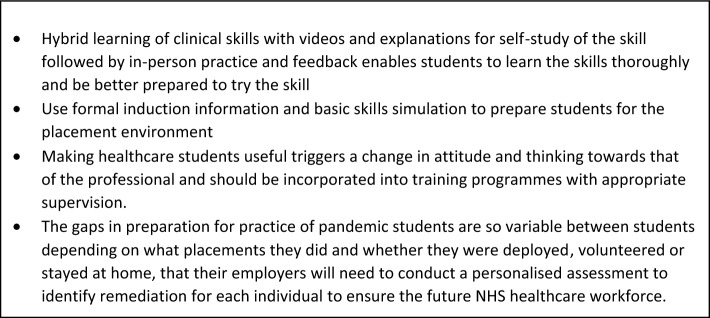
Recommendations for practice

These findings resulted in recommendations for future developments in clinical skills teaching and learning (Fig. 3).

The strengths of this study are its use of the realist approach to explain why various interventions worked or did not work for different groups of healthcare students. Drawing on the explanations of both students and their clinical supervisors and teachers has also strengthened the CMOcs.

The limitations of this study are evident when looking at the diversity of experience of healthcare students as described by our 51 participants. They were interviewed at the height of the pandemic and were unable to predict future outcomes which will require further study. In describing what seemed important to them they have shone a light into only parts of their professional courses and only parts of the local healthcare placements for a single UK University. There are doubtless other contexts which will have produced other outcomes in other parts of the UK and across the world. For institutions which have similar contexts, however, the same interventions are likely to trigger the same mechanisms with similar outcomes.

## Conclusion

To provide the NHS with a safe and effective workforce, it is important that we understand how effective the learning strategies employed during the COVID-19 pandemic have been and to understand across a broad spectrum of people, why they worked (or not). Our findings provide some useful context-specific explanations which can be drawn on in a future pandemic.

Successful innovations such as hybrid learning of skills and induction including virtual placements may be useful even after the pandemic in training the increasing numbers of healthcare professional students being recruited while the clinical workplace has limited placement capacity for them. Through understanding what strategies work for who, and why, it may be possible to train successfully in a more flexible working environment.

### Supplementary Information

Below is the link to the electronic supplementary material.

Supplementary file 1 (DOCX 23 KB)

Supplementary file 2 (XLSX 60 KB)
