# Supplementary material for: Training healthcare professionals to be ready for practice in an era of social distancing: a realist evaluation
Source: Adv Health Sci Educ Theory Pract. 2023 Dec 8;29(4):1265–84. doi: 10.1007/s10459-023-10297-w (PMC11369051; doi:10.1007/s10459-023-10297-w)
Supplement: Supplementary file 1 — (DOCX 23 KB) [file 10459_2023_10297_MOESM1_ESM.docx]

**Interview Topic guide**

**Research questions** (which can be shared with participants)

In a social distancing learning environment:

- What is working for students to promote their clinical readiness for practice, and why is it working?
- What has been tried and didn’t work and why?
- Where are the gaps in preparation for practice as yet not being filled in the educational system?

**Students:**

- Course, year
- How are you finding your clinical professional learning has changed since the COVID pandemic started?
  - What has worked for you to get you ready for clinical practice? (What teaching methods have the school used which have helped you most? What about placements has helped?)
  - How or why did it work?
  - What didn’t work for you? Why not?
    - How can this be improved?
- Do you feel like you are ‘missing out’ on any clinical learning?
- Have you been doing anything beyond the school’s planned learning to help to facilitate your clinical learning?
- Do you feel like online learning has influenced your readiness for your next clinical placement in any way?
  - Why?
  - How could this be improved?

**Staff:**

- What is your background (e.g. profession, qualification level)
- Experience / role in teaching
- How are you finding your clinical teaching has changed since the COVID pandemic started?
- What is working for your students to promote their clinical readiness for practice, and why is it working?
- What has been tried and didn’t work and why?
- Where are the gaps in preparation for practice as yet not being filled in the educational system?
- If doing any clinical teaching online:
  - How are you finding adjusting to teaching online?
  - What have been the barriers / facilitators?
  - Could you tell me about what you’ve been teaching online?
  - What methods have you used?
  - What do you think worked?
  - What do you think didn’t work?
    - How can this be improved?

(and these same questions can be asked for any other major change in clinical teaching)
